# Supplementary figures and images for: Littorally adaptive? Testing the link between habitat, morphology, and reproduction in the intertidal sculpin subfamily Oligocottinae (Pisces: Cottoidea)
Source: PeerJ. 2017 Aug 10;5:e3634. doi: 10.7717/peerj.3634 (PMC5554603; doi:10.7717/peerj.3634)

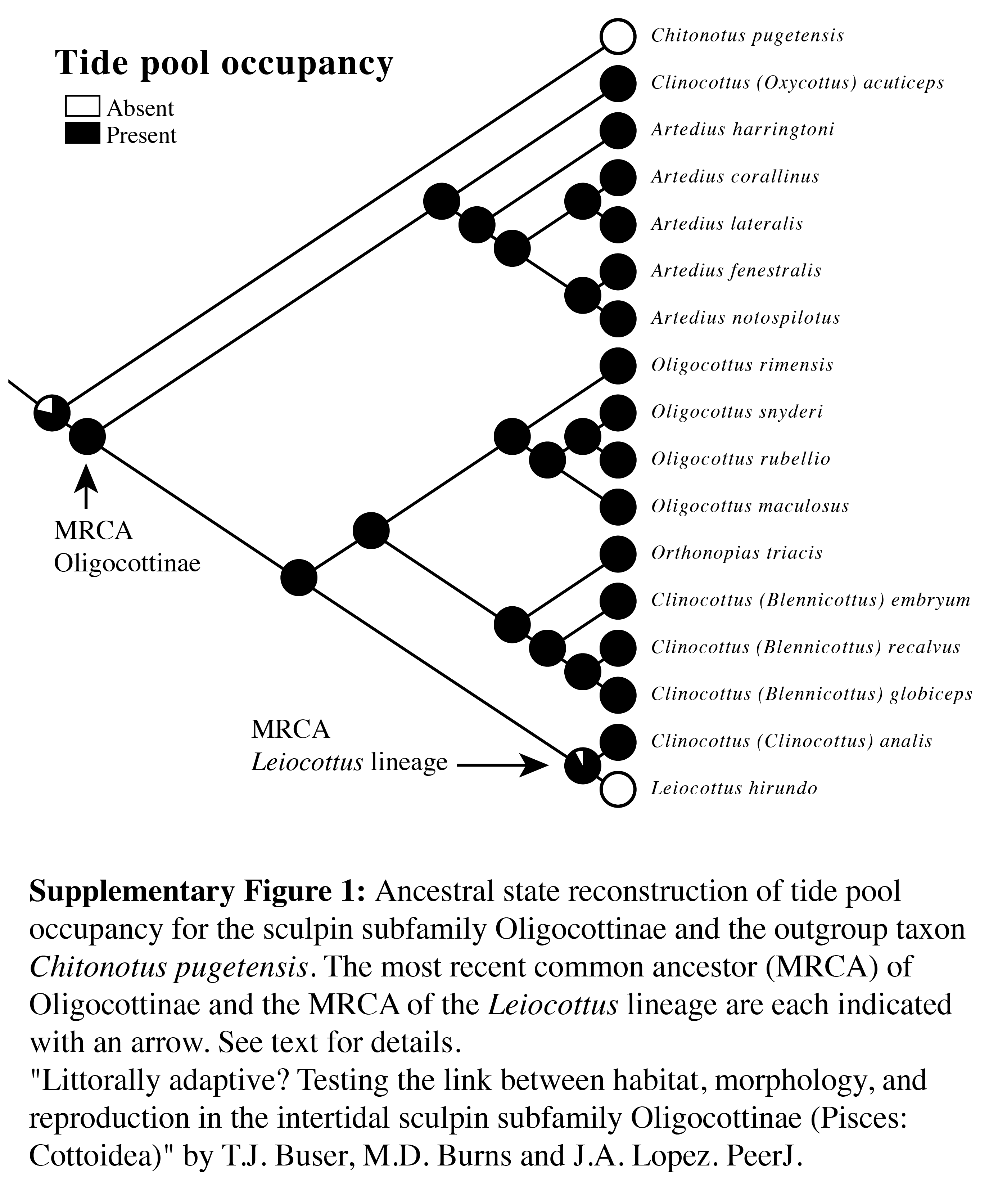

Supplement: Figure S1 — The most recent common ancestor (MRCA) of Oligocottinae and the MRCA of the Leiocottus lineage are each indicated with an arrow. See text for details. [file peerj-05-3634-s001.png]

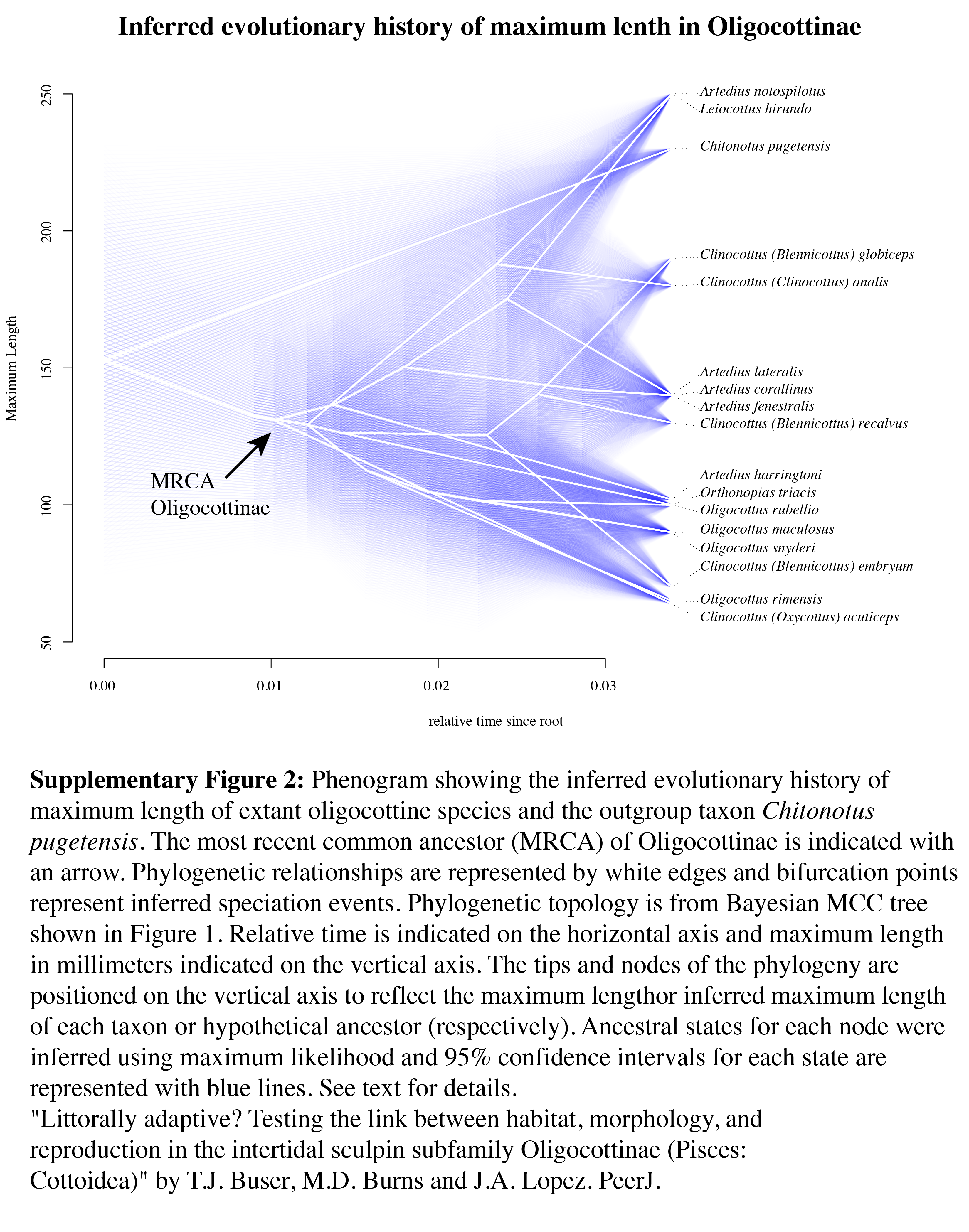

Supplement: Figure S2 — The most recent common ancestor (MRCA) of Oligocottinae is indicated with an arrow. Phylogenetic relationships are represented by white edges and bifurcation points represent inferred speciation events. Phylogenetic topology is from Bayesian MCC tree shown in Figure 1. Relative time is indicated on the horizontal axis and maximum length in millimeters indicated on the vertical axis. The tips and nodes of the phylogeny are positioned on the vertical axis to reflect the maximum length or inferred maximum length of each taxon or hypothetical ancestor (respectively). Ancestral states for each node were inferred using maximum likelihood and 95% confidence intervals for each state are represented with blue lines. See text for details. [file peerj-05-3634-s002.png]

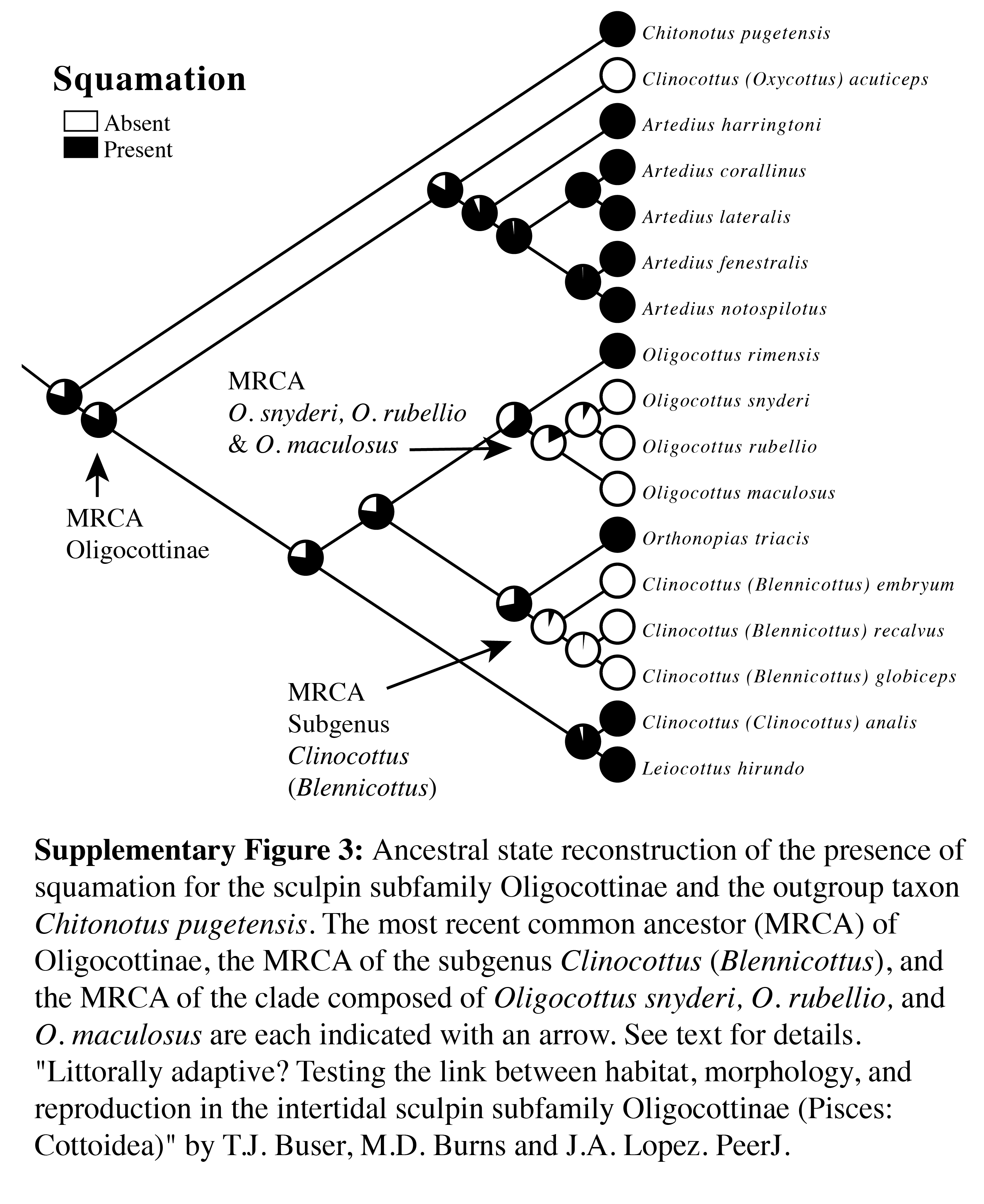

Supplement: Figure S3 — The most recent common ancestor (MRCA) of Oligocottinae, the MRCA of the subgenus Clinocottus (Blennicottus), and the MRCA of the clade composed of Oligocottus snyderi, O. rubellio, and O. maculosus are each indicated with an arrow. See text for details. [file peerj-05-3634-s003.png]

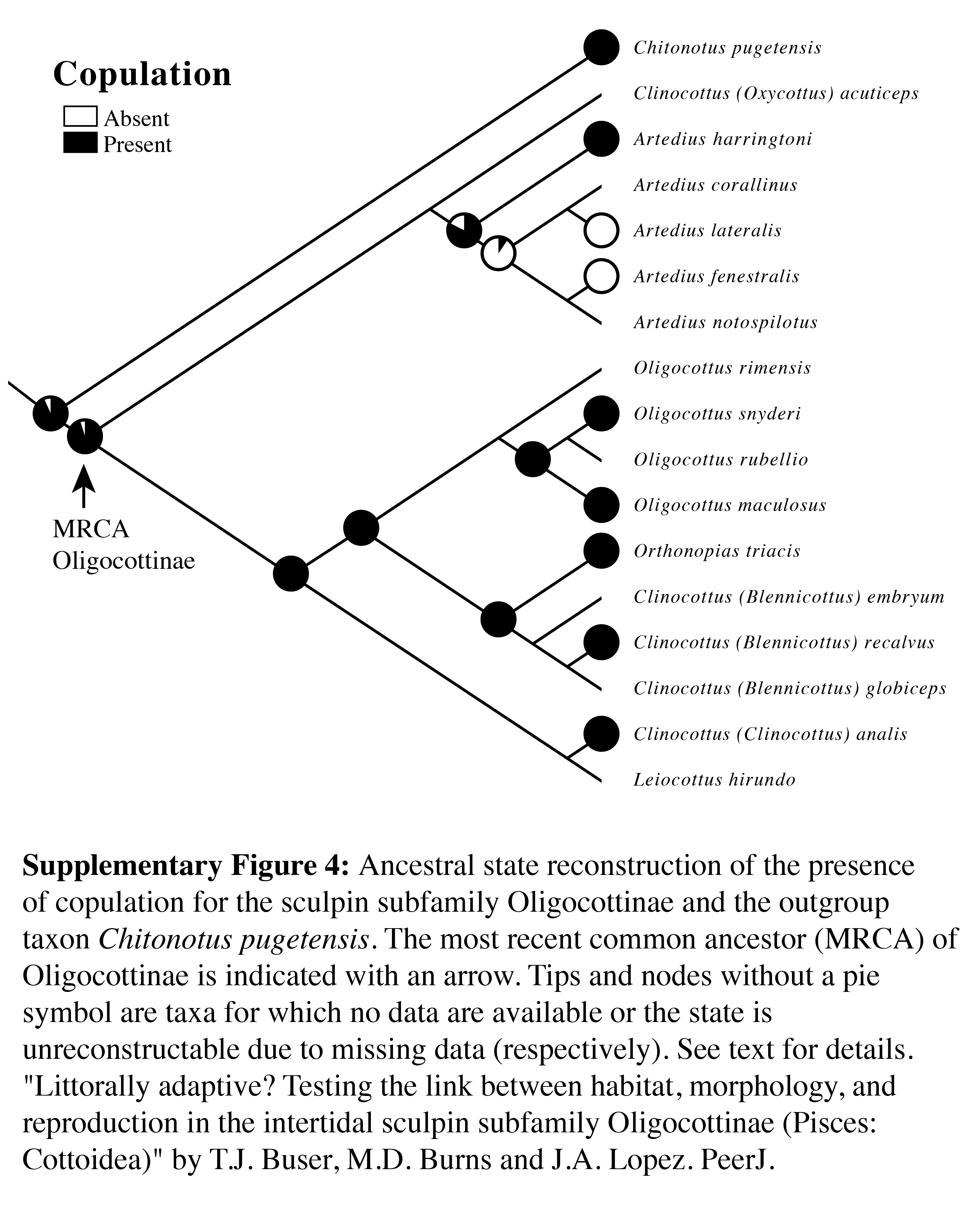

Supplement: Figure S4 — The most recent common ancestor (MRCA) of Oligocottinae is indicated with an arrow. Tips and nodes without a pie symbol are taxa for which no data are available or the state is not reconstructable due to missing data (respectively). See text for details. [file peerj-05-3634-s004.png]

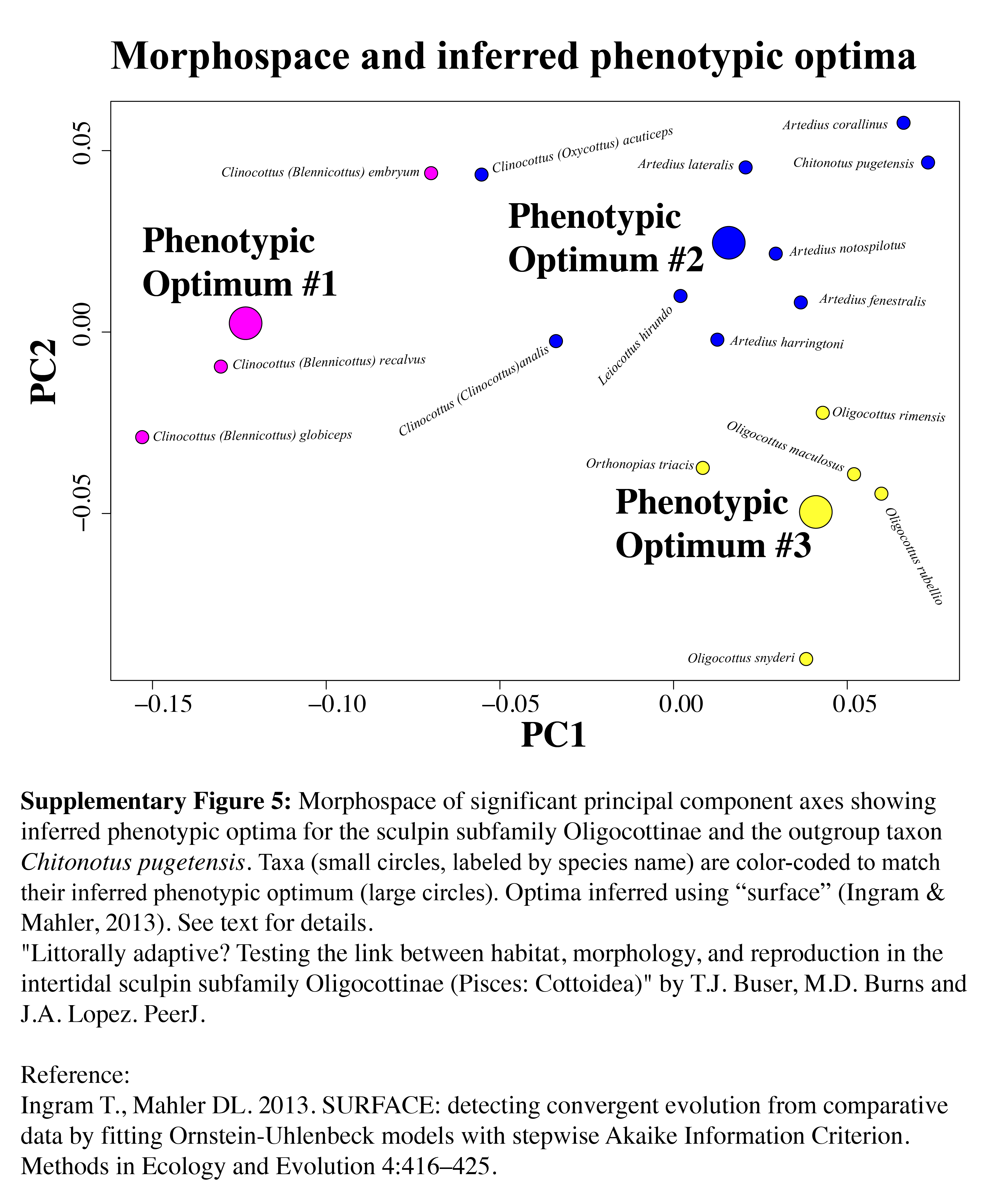

Supplement: Figure S5 — Taxa (small circles, labeled by species name) are color-coded to match their inferred phenotypic optimum (large circles). Optima inferred using “surface” (Ingram & Mahler, 2013). See text for details. Reference: Ingram T., Mahler DL. 2013. SURFACE: detecting convergent evolution from comparative data by fitting Ornstein-Uhlenbeck models with stepwise Akaike Information Criterion. Methods in Ecology and Evolution 4:416–425. [file peerj-05-3634-s005.png]

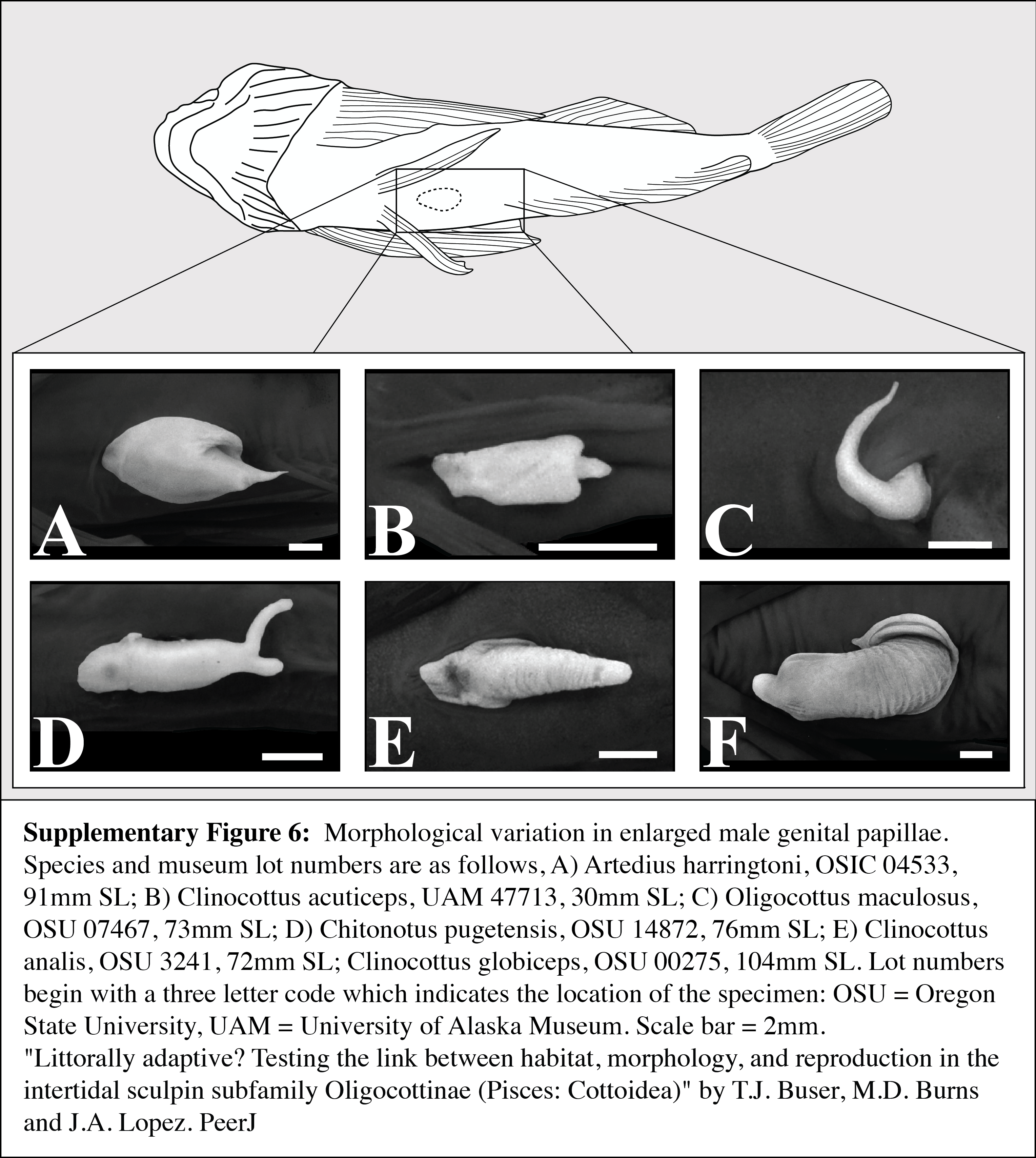

Supplement: Figure S6 — Species and museum lot numbers are as follows, (A) Artedius harringtoni, OSIC 04533, 91 mm SL; (B) Clinocottus acuticeps, UAM 47713, 30 mm SL; (C) Oligocottus maculosus, OSU 07467, 73 mm SL; (D) Chitonotus pugetensis, OSU 14872, 76 mm SL; (E) Clinocottus analis, OSU 3241, 72 mm SL; (F) Clinocottus globiceps, OSU 00275, 104 mm SL. Lot numbers begin with a three letter code which indicates the location of the specimen: OSU = Oregon State University, UAM = University of Alaska Museum. Scale bar = 2 mm. [file peerj-05-3634-s006.png]

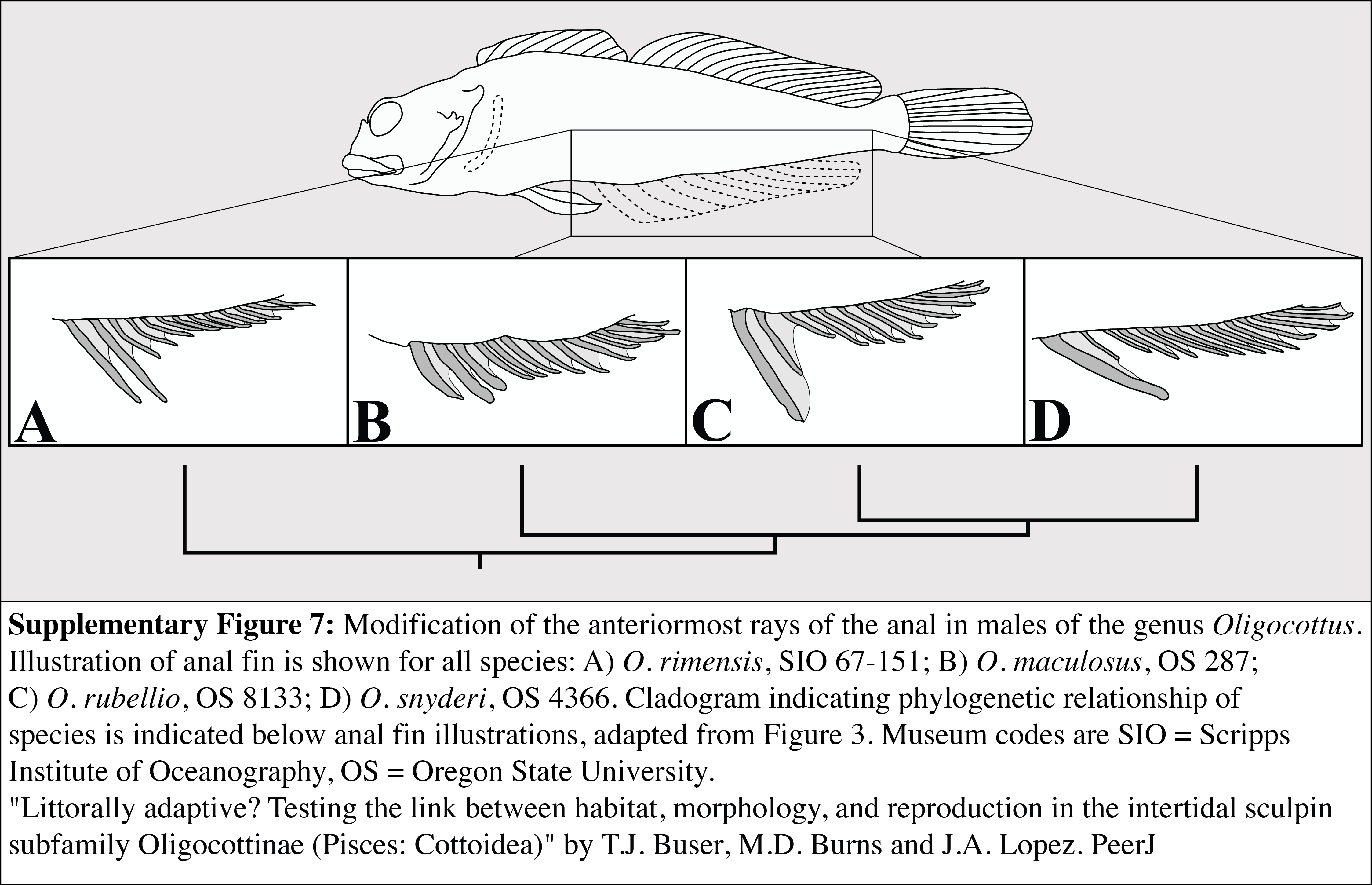

Supplement: Figure S7 — Illustration of anal fin is shown for all species: (A) O. rimensis, SIO 67-151; (B) O. maculosus, OS 287; (C) O. rubellio, OS 8133; (D) O. snyderi, OS 4366. Cladogram indicating phylogenetic relationship of species is indicated below anal fin illustrations, adapted from Figure 3. Museum codes are SIO = Scripps Institute of Oceanography, OS = Oregon State University. [file peerj-05-3634-s007.png]
